# Different phases of ATS use call for different interventions: a large qualitative study in Europe

**DOI:** 10.1186/s12954-022-00617-5

**Published:** 2022-04-12

**Authors:** Nienke Liebregts, Rafaela Rigoni, Benjamin Petruželka, Miroslav Barták, Magdalena Rowicka, Heike Zurhold, Katrin Schiffer

**Affiliations:** 1 Correlation-European Harm Reduction Network, Amsterdam, The Netherlands; 2 Department of Addictology, First Faculty of Medicine, Charles University, Prague, Czech Republic; 3 Institute of Psychology, Maria Grzegorzewska University, Warsaw, Poland; 4 Centre of Interdisciplinary Addiction Research of Hamburg University (ZIS), Department of Psychiatry, University Medical Centre Hamburg-Eppendorf, Hamburg, Germany; 5 Bonger Institute of Criminology, University of Amsterdam, Amsterdam, The Netherlands

**Keywords:** ATS use phases, Policy, Practice, Qualitative research, Prevention, Stimulants

## Abstract

**Background:**

Amphetamine-type stimulants (ATS) are globally widely used. Scientific literature generally defines four phases of substance use (initiation, continuation, increase and decrease); however, there is limited understanding of what influences these different phases of ATS use. The ATTUNE study investigated which factors shape individual phases of use, or ATS use patterns. In this article, we report on these phases into and out of ATS use, and propose a set of recommendations for prevention, harm reduction and treatment of the different phases of ATS use.

**Methods:**

Qualitative, semi-structured interviews (*n* = 237) were conducted in five different European countries with participants who had used ATS, varying from a few times in a lifetime to daily.

**Results:**

Amphetamine and MDMA were the most commonly used ATS. Yet, types of ATS used differed between the countries. We found that people who use ATS have various motives for and dynamic patterns of ATS use with alternating phases of increase, continuation, decrease and sometimes dependence. Cessation was pursued in different ways and for diverse reasons, such as mental health problems and maturing out. Availability seemed not an issue, regardless of the type of ATS, phase or country.

**Conclusions:**

These findings demonstrate that tailor-made interventions are needed for the diverse types of people who use ATS and different phases or patterns of ATS use, to reduce possible harms of use. We recommended a set of interventions for the different ATS phases. These include drug checking services, peer-led information, self-management of ATS use, mental health support to help people cope with stressful life events and prevent uncontrolled use, and follow-up support after treatment.

## Introduction

Amphetamine-type stimulants (ATS) (amphetamine, MDMA, methamphetamine, illicit use of prescription drugs, e.g. methylphenidate and Ritalin, and new psychoactive substances that mimic the effects of stimulants) are the second most commonly used illicit drugs worldwide and the third in Europe and create a large challenge in the synthetic drug area [1, 2]. Across the European Union, 1.2% of individuals aged 15–34 reported using amphetamines (amphetamine and/or methamphetamine) in the past 12 months and 1.9% MDMA. However, ATS use rates vary by country: the highest last-year prevalence for amphetamine among young adults aged 15–34 years is found in Germany (2.9%) and for MDMA in the Netherlands (6.9%) [1]. Amphetamine is rather prevalent in countries like Germany, Poland, Hungary or Norway, Sweden and Finland, and methamphetamine is mostly reported in Czech Republic, Slovakia and some Baltic states. MDMA use is found most popular in the Netherlands, Ireland, UK, Bulgaria [1].

Some qualitative and few quantitative studies exploring influences on ATS use have been conducted, primarily focusing on initiation of ATS use. A systematic review identified individual, social and environmental influences shaping key phases in ATS use: initiation, continuation, increase/relapse and decrease/abstinence [3]. Significant motives for the initiation of ATS use include curiosity [4–6]; to boost work/studies performance [7–9], endurance at dance events [9–11] and self-management of stress or mental health issues [5, 7, 12–14]. Stress and psychological distress have been found a complex factor for both initiation of ATS use, and continuation and increase in the use, in particular among people who use methamphetamine [15]. Some evidence suggests that continued and/or increased ATS use occurs to support specific functional needs (improvement of stress management or decreased insecurity in social situations) and to help cope with withdrawal effects [4, 11, 13, 16–19]. The experience of life events (new/ended relationships, death of a close family member, etc.) also appears to be associated with persistent use [7, 9, 18]. Factors that have been linked to a decrease in use include an increased perception of negative health impacts [5, 11, 13, 20], changing social networks and limited availability of ATS [21–23]. O’Donnell et al. [3] found that family, peers and social networks played a crucial role throughout all turning points: on the one hand, they facilitated access to ATS, and on the other hand, they supported normalization of ATS use over time [3]. Their review also showed that experiencing mental health problems, a relationship breakup and social and economic exclusion were returning themes involved in most phases of ATS consumption. Nonetheless, they showed that in general there is a lacuna in the current literature about what factors contribute to phases such as an increase and decrease in ATS use [3]. Long-term ATS use could lead to physical [24, 25], mental [26] and social harms (e.g. [27]) including (psychological) dependence (e.g. [28, 29]). However, there is limited understanding of what influences different phases of ATS use. Additionally, without thorough knowledge on what influences ATS use phases and its turning points, truly knowing what can prevent (further) harms in the different phases of ATS use, remains a blind spot as well.

Most interventions directed to prevent, treat or reduce the harms of illegal drug use currently focus on (injected) opioids [30, 31]. Yet, people who use ATS usually do not identify themselves with (problematic) opioid use, often belong to different networks of people who use drugs, and do not perceive opioid focused services as relevant to them [32]. They might develop different phases of drug use, face different drug-related harms and have different needs than those using opioids, thus requiring specific or adapted services [33]. Developing tailor-made and effective interventions for people who use ATS is necessary and requires a close analysis of the different groups, their specific needs and the relations that people establish with the different substances.

To fill these knowledge gaps on influencing factors on ATS use phases and subsequently on effective interventions, this paper builds upon the findings of a large multinational research (ATTUNE) on ATS phases. Based on ATTUNE’s qualitative data of ATS phases as described by people who use ATS in five European countries—the Netherlands, UK, Germany, Poland and Czech Republic—our paper proposes a set of recommendations for prevention, harm reduction and treatment of the different phases of ATS use.

## Methods and data

### Study design

ATTUNE was a multinational study that investigated which factors shape individual ATS use phases (see [34]). The study was conducted in the Netherlands, UK, Germany, Poland and Czech Republic. A sequential, exploratory design was used, combining quantitative and qualitative methods. For purposes of this paper, we focus on the qualitative data only, namely the in-depth interviews conducted with people at different phases of ATS use. Exposed people who do not use ATS were also included in the study but left out for this paper, since we focused on actual use and interventions. Qualitative research allows exploring beliefs, perspectives and experiences of people who use ATS that influence trajectories of substance use, also improving our understanding of the processes and social context involved in changes [35, 36]. For prevention, harm reduction and treatment programmes such in-depth insights are deemed essential [3, 36].

### Participant groups and recruitment

To reach a sufficient variety of ATS use patterns, we divided participants according to their currency and frequency of ATS use. The qualitative interviews targeted five different groups, each with equal size and gender distribution: currently dependent use (CDU, group 1), formerly dependent use (FDU, group 2), non-dependent current frequent use (CNU, group 3), formerly frequent use (FNU, group 4) and non-frequent use (NNU, group 5). Current use referred to ATS use in the past 12 months. Frequent use was defined as > 10 consumption days in 12 months. Dependence on ATS was assessed using the severity of dependence scale (SDS) [37] with the recommended cut-off for ATS of ≥ 4 points [38].

Individuals who had used ATS were regarded as eligible for inclusion in the ATTUNE study. Participant’s first ATS use had to take place at least 5 years before the interview to ensure inclusion of only those who had had the opportunity to experience changes in their ATS use career. To avoid overlap between trajectories into opioid use and trajectories into ATS use, people previously diagnosed with opioid dependence (self-reported) were excluded.^1^ This also prevented domination of the sample by people who primarily use(d) opioid and use(d) stimulants only to complement their opioid use. Other inclusion criteria were: aged ≥ 18 years; being a resident in one of the five national sampling regions; and verbal and cognitive ability to take part in the interview.

Multiple purposive sampling strategies were used to recruit participants into the study, including snowball sampling; announcements on social media and internet forums; leaflets and posters distributed at universities, student portals, nightlife settings and third-sector organizations; via gatekeepers and professionals at substance use services and treatment facilities; and via the researchers’ social networks. The sampling criteria were monitored for the different groups, as well as diversity regarding age, gender and treatment experiences. Not in all countries, participants were evenly distributed over the five groups, as some profiles were harder to find than others.

### Data collection and analysis

Interviews were conducted face-to-face between February and August 2017. Each interview lasted on average 1 h. They took place at different (quiet) locations, mostly at the university, research institute, at participant’s house or in public spaces such as a cafe. After completion of the interview, each participant received an incentive (money or vouchers, depending on the country).

A guideline was used to structure the interviews. It included questions about participants’ ATS use career (i.e. changes in use trajectories and occurrence of life events in various domains). Interviewees were asked to recall changes in different life domains and their ATS use patterns in their entire life, using individual time charts [39, 40]. The time chart referred to the frequency of substances used and to experiences in life domains (including social life, health, work/study, leisure). All interviews were digitally recorded (with participant’s consent), fully transcribed and entered into NVivo or MaxQDA. The analysis of the interviews was structured around the four phases of use (initiation, continuation, increase and decrease) which were based on the literature [3]. For each of the phases, the individual, social and environmental circumstances were explored. Each country analysed their own data through a similar detailed coding scheme and wrote a report with the findings, which were then combined and compared for all countries on main patterns and differences. To guarantee anonymity, interviewees were identified with an alias (name or number).

## Results

### Participants

In total, 237 persons who use(d) ATS were interviewed in the five countries, (unevenly) distributed over the target groups (see Table 1). In total, 41% were female and participants were on average almost 32 years old (31.7 years). Overall, participants from group 3 (CNU) were the youngest with a mean age of approximately 29 years, while the respondents from group 2 (FDU) were the oldest ones with a mean age of over 33 years. Overall, ATS use was initiated at age 17–19 years (Table 1). The lowest mean age of onset was found in group 1 (CDU, 17.3 years) and group 4 (FFU, 17.4 years) and the highest age of onset in group 5 (NNU, 19.0 years). More than one-third of the total sample scored positive on the severity of dependence scale, indicating ATS dependence. The vast majority of ATS-dependent interviewees were from group 1 and 2 (CDU and FDU, as targeted).

**Table 1:** Age, age of ATS onset and ATS dependence of participants by country and target groups

Country	Mean	Group 1 CDU	Group 2 FDU	Group 3 CFU	Group 4 FFU	Group 5 NNU	Total
Germany	Total *n*	9	17	12	6	9	53
	Female *n*	5	8	3	2	3	21
	Age (range)	28.2 (18–37)	30.2 (24–37)	27.2 (21–32)	34.7 (20–63)	33.8 (22–53)	30.8 (18–63)
	Age of ATS onset	16.3	17.6	18.3	21.2	21.8	18.7
	SDS positive %	100	88.2	16.7	0	0	48.3
UK	Total *n*	12	14	9	11	11	57
	Female *n*	7	6	4	8	5	30
	Age (range)	37.2 (21–52)	35.4 (23–62)	32.6 (19–50)	30.5 (23–42)	28.1 (20–40)	32.8 (19–62)
	Age of ATS onset	18.0	16.2	18.2	15.8	17.2	17.0
	SDS positive %	100	85.7	11.1	18.2	18.2	42.6
Poland	Total *n*	10	10	12	5	15	52
	Female *n*	3	5	1	2	4	15
	Age (range)	30.1 (18–42)	32.7 (23–54)	25.3 (23–34)	33.2 (19–42)	33.3 (23–40)	30.9 (18–54)
	Age of ATS onset	18.1	17.3	17.7	16.4	18.1	17.7
	SDS positive %	60.0	70.0	0	0	0	21.3
The Netherlands	Total *n*	10	10	10	10	10	50
	Female *n*	5	4	4	4	6	23
	Age (range)	34.9 (26–43)	35.2 (23–57)	31.0 (22–44)	35.9 (26–55)	28.9 (22–34)	33.2 (25–36)
	Age of ATS onset	16.4	17.6	19.3	17.3	19.8	18.1
	SDS positive %	80.0	100	0	0	0	30.0
Czech Republic	Total *n*	6	5	5	5	4	25
	Female *n*	2	2	0	1	2	7
	Age (range)	26.8 (20–37)	38.2 (20–54)	34.6 (24–47)	27.4 (21–31)	29.5 (26–33)	31.3 (20–54)
	Age of ATS onset	17.5	26.8	23.0	17.8	19.5	20.8
	SDS positive %	83.3	100	0	0	0	33.3
Total	Total *n*	47	56	48	37	49	237
	Female *n*	22	25	12	17	20	96
	Age	32.1	33.6	29.3	32.6	31.0	31.7
	Age of ATS onset	17.3	18.0	18.8	17.4	19.0	18.2
	SDS positive %	85.1	85.7	6.3	16.2	4.1	35.5

There were some notable differences between the five groups and the countries. In the Netherlands, an equal gender distribution was realized as intended, while in Germany, Poland and the Czech Republic more males than females were interviewed. In the UK, more females participated in the interviews. The average age of participants was lower in Germany (30.8) and higher in the Netherlands (33.2). Regarding age of onset, some groups per country stand out: the lowest age of onset (15.8 years) was found in group 4 (FFU) in the UK and the highest in group 2 (FDU) in the Czech Republic. The proportion of respondents with ATS dependence varied across the countries from 21% in Poland to 48% in Germany. Especially in Czech Republic, the Netherlands and the UK, between 70 and 100% of the group 1 and group 2 members (CDU, FDU) had ever used any ATS daily. Daily ATS use (lifetime) participants (in groups 1 and 2 CDU, FDU) had been in contact with drug counselling services or addiction treatment at least once, ranging from drug counselling, detoxification, to residential drug treatment.

Types of ATS used differed between the countries (not in table). In the Czech Republic, methamphetamine was most prevalent (used by 60% of participants). In all other countries, methamphetamine was less common. In Germany, the use of this substance only occurred in the border region. Types of ATS ever used also differed across the groups (Table 2). Across all groups, amphetamine and MDMA had ever been used by the far majority. Slightly less of the members of group 5 (NNU) had ever used these ATS, yet still around two-thirds had used them. Methamphetamine was ever used by approximately one in three participants, but only a few of them were found in group 5 (NNU). ATS-like NPS were also used by 1 in 3 participants, but of the group 3 members (CFU), almost half of them had ever used this ATS.

**Table 2:** Types of ATS used lifetime across the five groups

	Group 1 CDU currently dependent users (*n* = 47)	Group 2 FDU formerly dependent users (*n* = 55)	Group 3 CFU non-dependent current frequent users (*n* = 48)	Group 4 FFU formerly frequent users (*n* = 37)	Group 5 NNU non-frequent users (*n* = 49)	Total (*n* = 237)
Amphetamine lifetime (*n* = 234)	42	44	40	32	32	190
MDMA lifetime (*n* = 224)	34	44	40	32	34	184
Methamphetamine lifetime (*n* = 228)	17	22	11	12	5	67
ATS-like NPS lifetime (*n* = 224)	16	14	23	8	13	74

### ATS use phases

#### Initiation

Participants motivated their first ATS use predominately by either hedonism or coping with difficult personal situations and/or mental health problems. Hedonism implied curiosity, pleasure-seeking and the desire to stay awake on party weekends. Coping was related to suppressing or managing experiences of depression, low self-esteem or social phobia. Few interviewees initiated ATS use for functional reasons to enhance work or studies performance or to increase sexual pleasure. In particular, students used methylphenidate (e.g. Ritalin) for a limited period of time to enhance performance in their studies. Furthermore, several participants linked their first ATS use to peer influences. ATS using peers were important to ensure availability at initiation, but also to inform about dosage and effects of the substance. While several respondents described the availability of the preferred ATS substance as a challenge, in the beginning, costs were not an issue in the phase of initiation.

At initiation, most participants mentioned the positive effects of the substances such as feeling alert, energetic and without hunger (amphetamine), feeling socially connected, talkative and happy (MDMA). The effects of methamphetamine were experienced as ambiguous due to an extraordinary feeling of well-being, on the one hand, and the loss of concentration as well as feelings of paranoia, on the other hand. People who used methamphetamine tended to increase their frequency of use rapidly (compared to other ATS) and to become dependent after a short period of time. These interviewees who used methamphetamine were often found in group 1 and 2 (CDU and FDU). The other groups showed no specific patterns regarding initiation.

#### Continuation

Continuation generally referred to the phase after initiating ATS and in some cases to the period after increased or decreased ATS use. The main use pattern in this phase was relatively stable and did not indicate a clear increase or decrease. The core emphasis of participants in this phase was wanting to preserve the positive effects from ATS, using ATS use for pleasure and to occasionally escape their ‘normal’ life routine. Continuation was also ascribed to the rather normalcy of ATS in participants’ social network. For some participants, the continuation phase was related to functional use, for example, to cope with demands of everyday life (manage household, work), to feel more energetic or to lose weight (amphetamine). Regarding the different groups, predominately those with frequent user (group 3, CNU) and non-frequent use (group 5, NNU) continued their ATS use at a more or less rather stable pace, indicating self-regulation. As part of their lifestyle, they (regularly) used ATS use on weekend nights out.

#### Increase and dependence

The stories of the interviewees showed a fine line between continuation and increase, and for many, this went hand in hand, especially when referring to the phase after initiation. A rather common pattern was an increase in use frequency after the first couple of times using ATS. Associating more frequently with others who used ATS often went together with increased use. Group 3 and 4 members (CFU, FNU) reported increasing ATS use because of its effects in the party scene and also related it to becoming more experienced with use, getting more familiar with its effects and combining ATS with other drugs.

For part of the participants, the increased use led to problematic and/or to dependent use (according to the SDS) and the line between non-problematic and problematic use was not always clear. Generally, their patterns of use developed slowly from recreational to very frequent use, into the direction of problematic use, and/or dependence. Many participants who reported to experience problematic use or who were dependent on ATS (according to the SDS, in group 1 (CDU) and 2 (FDU)) linked their use patterns to underlying mental health problems, negative emotions, lack of self-confidence or a boring job. They often maintained frequent ATS consumption for several years.

In the phase of increased frequent or excessive ATS use, side effects such as sleeping disorders, weight loss, memory loss and concentration problems were commonly reported. Other health issues included intensified feelings of depression, anxiety or paranoia. Many participants who frequently used ATS reported using cannabis or other substances to come down from the ATS effects, to be able to sleep and be fit for the week ahead. For some interviewees, their increased use negatively impacted daily responsibilities such as work, studies or childcare, or their social relationships: interactions with people who do not use ATS were avoided.

ATS were generally easily available in the increase phase. Costs became challenging for some participants: while ATS were usually considered as rather affordable, dependent use meant higher costs and potential difficulties affording it. Few of these participants financed their use through debts, selling personal items, loan sharks or (social) dealing.

While most interviewees with problematic use were by definition found in group 1 (CDU) and 2 (FDU), also a few group 3 (CNU) and 4 members (FNU) reported to have experienced problematic (past) or compulsive use. Group 1 (CDU) and 2 (FDU) participants who used methamphetamine contrasted with other groups in the increase phase who gradually increased their use: they generally increased their use rapidly towards daily use. Group 1 (CDU) and 2 (FDU) participants who frequently used amphetamine commonly reported drinking amounts of alcohol, and some of them developed alcohol dependence, which from their perspective was their major problem.

#### Decrease and desistance

Decrease signified a stage when ATS use clearly shifted into non-frequent use, self-regulated use or abstinence from ATS. Almost always, in the participants’ perspective decreased use referred to a lower frequency of use with reference to previous use. The length and underlying motives for decreased use were very varying from intended to unintended, from a short period to a longer time and from focusing on daily priorities to an unsuccessful attempt to use less often.

By definition, participants from group 4 (FNU) had already stopped their ATS use at the time of the interview. Yet many participants from the other groups also reported a period of decreased ATS use, which could result in desistance from ATS. Whereas participants generally referred to a lessened frequency of use, mainly some group 1 and 2 members (CDU, FDU) specifically mentioned less quantity/smaller doses when talking about decreased use.

Among participants who were dependent and some who frequently used ATS (CDU, FDU, CNU), side effects of ATS use and related health problems most often led to the decision to decrease or quit. These included severe physical exhaustion, panic attacks, blackouts, depression, lack of appetite, paranoia and dental problems. For some interviewees, mainly ATS-dependent participants [group 1 (CDU) and 2 (FDU)], (mental) health problems had led to the decision to enter treatment. Rather often treatment was also used for alcohol problems partly related to excessive amphetamine use. Especially for methamphetamine-dependent interviewees drug treatment became essential for their recovery. Some participants felt that the accessed treatment services were not sufficiently focused on ATS use. However, rather often treatment entries were also related to mental health disorders and multiple stressors around unemployment, poverty or domestic violence. In particular, females reported multiple stressors which led to difficulty in quitting ATS without professional support.

Another commonly found pattern in decrease and desistance of ATS was gradually maturing out, particularly found in group 3 (CNU). Responsibilities and priorities such as a new partner or a new job became more important, and/or nightlife considered boring after a while. Moreover, most of the women with children abstained from ATS during pregnancy, although some started reusing once their child was born. When reflecting on their ATS career during the interview, some interviewees from group 3, 4 and 5 (CNU, FNU, NNU) realized that they never made a hard decision regarding their ATS use, but that it gradually had been moved to the background of their daily life. Some of them had desisted from ATS, others used ATS only irregularly. For others, the phase of decrease or abstinence was challenging, especially the reorganization of priorities in life. They focused on studies and more often associated with people who do not use ATS (frequently) (hedonistic type), or focused on parental responsibilities (women with children) or more generally on re-establishing daily routines, such as moving back to former hobbies or sports or finding new leisure interests. Particularly for dependent participants reorganizing daily life and keeping distance to ATS-using environments was generally difficult. Some of them moved to another city, ended romantic relationships or avoided certain geographical areas. For a few ATS-dependent interviewees, imprisonment enforced abstinence.

## Discussion

Our study showed that phases of ATS use and the people who use it are diverse. People who use ATS have divergent motives for use, dynamic patterns of use with alternating phases of increase, continuation and decrease and different ways of and reasons for cessation. This heterogeneity is also a result of the diverse group of participants that we recruited by using a variety of recruitment strategies.

At initiation, use is often motivated by curiosity and pleasure-seeking, and sometimes to improve performance at work/studies or to cope with mental health problems. This is in line with previous studies (see for example [5, 6]). An increase in ATS use is often associated with an increasing orientation towards a drug-using environment (party lifestyle) or individual and social stressors such as a relationship breakup or mental issues (cf. [5, 15]). Occasional, controlled use was practised by participants who prioritized everyday commitments and who used them on selected occasions. We also found mental health problems involved in most phases (cf. [3]). Decrease or eventual cessation of ATS use was associated with experiencing serious health effects of use and increased stress from neglecting work, family and relationships. Many but not all of those with dependent or problematic ATS use had been in counselling and treatment. In contrast, people who used ATS primarily at parties or during nightlife often matured out of ATS use. Thus, for some, desistance or decrease phases were induced by turning points such as imprisonment or a new job, yet for others there never had been a conscious decision to quit. Availability seemed not an issue, regardless of the type of person who used ATS, phase or country, while previous studies linked limited availability of particularly methamphetamine to decrease [21].

ATS use, in its different phases, may bring a variety of harms for people who use these substances. An increasing body of studies has analysed and proposed prevention, treatment and harm reduction interventions for people who use ATS. Studies usually investigate the effectiveness of interventions for a specific substance, or form of administration, and pay less attention to the phase of ATS use in which such interventions can be beneficial. By combining the findings of our study with this scientific literature, we propose evidence-based interventions which can be beneficial to reduce the harms of ATS use in the different phases of ATS use.

### Evidence-based information

Evidence-based information for people who use ATS about substances and their effects, and how to reduce potential harms of ATS use can be beneficial in all phases of ATS use. Important aspects to consider which information to provide are the motivations of specific groups for using the substance as well as individuals’ perceived risks associated with use [41]. Other important information includes the potential consequences of mixing ATS with other drugs, including alcohol [42], the possibility of engaging in high-risk sexual behaviours [43] and educating people who use ATS about potential sleeping problems as well as hyperthermia [44]. Online interventions providing information to prevent (continued) MDMA and methamphetamine use, and harms, were also found to be effective [45].

### Peer-based interventions

Several ATTUNE participants linked peers to their initiation, continuation and decrease in ATS use. Peer-based programmes can be very effective, as information and knowledge is experience-based and can contribute to the credibility of the intervention (e.g. [46]). Peers with experience of ATS use and preferably, part of the same sub-groups of people who use ATS for whom the intervention is planned, should be meaningfully involved in the design and the provision of different interventions and phases of ATS use. For instance, peers have been crucial in planning for and distributing safer smoking kits for people smoking methamphetamine [47] helping minimize related harms. They have also effectively engaged with those using MDMA in party settings [48] to bring much needed information on safer drug use. Peers can also offer counselling for supported withdrawal, including providing information around the withdrawal process for those willing to quit ATS use, helping to identify protective and risky factors in previous withdrawals and helping identify key social supports [49].

### Primary healthcare responses

Primary health care can be instrumental in responding to both direct and indirect consequences of regular stimulant use. There are usually located close to communities and represent a low threshold point of care. When accessible to people who use drugs without judgement or stigma, primary health care can help preventing acute health problems from worsening and developing into chronic conditions, for instance, among young people (e.g. [50]). They can also be crucial to provide care for more vulnerable communities such as indigenous people [51] or women [52] using methamphetamine if welcoming the specific cultures and needs of these populations.

### Self-management of drug use

People who use drugs, including those using various types of ATS, are often able to control their drug use in varying levels of success [53]. Self-management of drug use can lead to less problematic patterns of use [54] and increases the chances of becoming and staying abstinent of drugs [55]. People who use stimulant drugs often create (informal) rules to self-manage their use according to perceived risk and triggers, such as only using when feeling well, using only with friends or during weekends and establishing a maximum amount or frequency of use [56]. While self-management can be learned, and supported by peers, it must build upon the person’s ability, empowering the skills and competencies they already use to control their use and reduce their risks [57].

### Mental health support

Several people who use ATS do so to cope with difficulties and existing mental health problems [13]. Frequent ATS use may also lead to mental harms such as depression, psychotic symptoms (hallucinations) and paranoid thoughts [26]. Moreover, chronic use is associated with high levels of psychiatric comorbidity (as depression, PTSD, ADHD, eating disorders and suicidal thoughts/attempts) [27]. Mental health support, thus, can be used in initial phases to help people cope with stressful life events and prevent increased/uncontrolled use [58], or in dependent or continued trajectories, to help tackling the mental harms (partly) due to extended drug use. The connection between drug use and mental health disorder is complex and an integrated approach is needed.

### Drug checking services and nightlife services

The illegal status of ATS often leads to unknown dosages and contents, increasing the risk of overdose as well as of other harms. In this context, drug checking and nightlife services can help to detect adulterants in substances, which can decrease someone’s intent to consume potentially dangerous substances and help inform harm reduction efforts. These services can also be crucial for issuing preventative warnings (in case of dangerous adulterants), helping to avoid further harm. Nevertheless, drug checking alone might not be sufficient: in particular, people who use ATS less frequently may require education about adulteration and drug checking, and referral to support services and drug education are important facilitators of harm reduction intentions [59].

### Safer social settings

Interventions that are placed in and adapted to party settings can be very useful to engage people who use ATS in reducing harms, especially the ones in the initial phases of ATS consumption, but also those continuing or increasing use. Chill-out rooms at festivals or in clubs, for instance, can help people who use MDMA to increase their fluid intake and prevent hyperthermia, as well as warn people of the potential harm of overconsumption of fluids [60]. Other practices include temperature control at the party venue, with adequate ventilation; provision of free cold water; staff training to understand and manage drug-related risks and emergencies; and adequate emergency provision [61].

### Pharmacological treatment

Medications can be useful in the management of stimulant intoxication, withdrawal and dependence. Benzodiazepines, for instance, have shown to be effective in managing intoxication of stimulants as well as (some) withdrawal symptoms [62]. Agonist or ‘substitution’ therapy involves the use of medications to counteract the dopaminergic deficit in people suffering from abstinence of stimulants. While agonist therapy has been widely acknowledged as effective for opioid, alcohol and tobacco dependence, there is limited evidence of its benefits for ATS. While high-quality controlled studies are limited in number, a recent systematic review [63] and randomized control trials [64, 65] point to accumulating evidence that psychostimulant medications can help people who use stimulants achieve and maintain abstinence and/or increase drug treatment retention. So far, studies have demonstrated limited benefits for drugs such as methylphenidate, bupropion, modafinil and naltrexone [66]. Pilots using prescription psychostimulants for methamphetamine dependence are in place in Czech Republic [67], and further research is needed to confirm benefits.

### Abstinence-based treatment and counselling

For those who are dependent on ATS and/or are willing to quit using, abstinence-based treatment and supportive counselling can be recommended. A few specific abstinence-based treatments have been developed for ATS, such as the Matrix model [68]. A specific structured brief counselling has been developed for people who regularly use methamphetamine, and has proved to help increased abstinence, and manage the risks of tobacco smoking, polydrug use, risky injecting behaviour, criminal activity and psychiatric distress [69]. Brief interventions have also shown to help reducing MDMA use and severity of MDMA-related problems in people with non-dependent but frequent use [70] and promoting readiness to change [71]. Motivational interviewing has also been effectively used to address low motivation to cease methamphetamine use and increase treatment access [72]. In any treatment chosen, follow-up after treatment completion is crucial.

### Limitations and future research

There were some methodological differences between the countries regarding sample size, incentives given and recruitment procedures. These methodological differences could have affected the final creation of the sample and with that the comparability of the data between the countries. While we did not structurally compare countries, and while countries probably have yielded different types of ATS use and prevalence, findings between the countries were largely in accordance. Neither did we perform analyses of longitudinal patterns of use, i.e. change between the different phases. For the analysis no (true) distinctions were made between the type of ATS. Despite being aware of the differences between the different ATS substances and people who use ATS, this procedure was chosen for emphasizing the phases of use. Finally, this paper is based on qualitative data. Our main aim was to describe phases of ATS use in five European countries and connect a set of recommendations for prevention, harm reduction and treatment to the main patterns of these phases. While we believe our findings brought valuable insights on patterns of ATS use, it must be noted that our findings per group are not based on statistically significant differences. The numbers of participants per group are rather small for quantitative analyses, and we encourage other scholars to explore ATS use phases with larger sample sizes.

For future research, it might be interesting to focus more on motives and processes of decreasing ATS use. In particular, studies which consider barriers and supportive factors within the phase of reducing or desistance of ATS use would contribute to the current lack of knowledge. Furthermore, it would be of major interest to learn more about the effectiveness of drug and alcohol treatment for people who use ATS, as services usually address drugs and alcohol problems in general, but our study showed that among dependent ATS participants these often go along. Finally, an effect evaluation of the proposed set of interventions would be very beneficial regarding harm reduction for the different phases of ATS use.

## Conclusions

This paper is among the first to give in-depth insights into phases of ATS use and connect tailored interventions to them, offering for practice, prevention and policy. Our study showed that the persons using ATS and their phases of use are diverse. They may have divergent motives for use, dynamic patterns of use with alternating phases of increase, continuation and decrease and different ways of and reasons for cessation. These findings demonstrate that tailor-made interventions are needed, to reduce possible harms of use. Based on the study findings and previous (evidence-based) studies and interventions, we recommended a set of interventions for the different phases of ATS use. These include drug checking services, peer-led information and education, self-management of ATS use, mental health support to help people cope with stressful life events and prevent uncontrolled use, and follow-up support after treatment.

## Data Availability

Due to the nature of this research, participants of this study did not agree for their data to be shared publicly, so supporting data is not available.

## Footnotes

1 Not for UK.
